# Differential Risk of Incident Fractures Depending on Intensity and Frequency of Physical Activity According to Cognitive Status: A Nationwide Longitudinal Study

**DOI:** 10.3389/fmed.2020.572466

**Published:** 2020-12-08

**Authors:** Dong Woo Kang, Sheng-Min Wang, Yoo Hyun Um, Hae-Ran Na, Nak-Young Kim, Kyungdo Han, Chang Uk Lee, Hyun Kook Lim

**Affiliations:** ^1^Department of Psychiatry, College of Medicine, Seoul St. Mary's Hospital, The Catholic University of Korea, Seoul, South Korea; ^2^Department of Psychiatry, College of Medicine, Yeouido St. Mary's Hospital, The Catholic University of Korea, Seoul, South Korea; ^3^Department of Psychiatry, College of Medicine, St. Vincent's Hospital, The Catholic University of Korea, Suwon, South Korea; ^4^Department of Statistics and Actuarial Science, Soongsil University, Seoul, South Korea

**Keywords:** fracture, dementia, subjective cognitive decline, older adults, physical activity

## Abstract

**Background:** Previous studies have demonstrated an increased risk of fractures in subjects with various degrees of cognitive impairments. Recently, there has been growing recognition of the vital effect of physical activity (PA) on delay and prevention of fractures in older adults.

**Objectives:** This study aimed to evaluate the optimal intensity and frequency of PA needed to prevent fractures in cognitively preserved older adults (CP), participants with subjective cognitive decline (SCD), and dementia patients using a large-scale nationwide cohort study.

**Methods:** Data from a nationwide health screening program for individuals at the transitional age of 66 years were used in this study. A total of 968,240 subjects was enrolled, followed from 2007 to 2014, and classified as CP (*n* = 759,874), SCD (*n* = 195,365), or dementia group (*n* = 13,001). Adjusted hazard ratios (aHRs) by demographic and known risk factors for fractures were evaluated to identify the impact of various frequency and intensity PA on the occurrence of hip, vertebral, and limb fractures.

**Results:** In CP participants, the most noticeable reduction of hip and vertebral fracture risk was shown in those performing vigorous-intensity PA at least three times per week. In the SCD group, the risk decrement in hip and vertebral fractures was most prominent in subjects who performed multiple-intensity PAs at least three times a week regardless of intensity. In the dementia group, only high-frequency walking and high-frequency & multiple-intensity PA decreased the risk of hip fractures compared with absence of PA.

**Conclusion:** These findings suggest a role for various PA intensity and frequency levels to prevent hip and vertebral fractures according to cognitive status. Further study is needed to validate the effects of PA intensity and frequency proposed in this study on fractures according to cognitive status.

## Introduction

Falls are highly common in older adults and occur at least once a year in ~30% of this population (1). Fractures are one of the main consequences of falls and impose extra risk to older adult health (2). Fractures are a major risk for disability (3), and less than half of older adults can perform independent activities of daily living up to 2 months after a fracture (4). Pelvic and vertebral fractures have been recognized as risk factors for death in the older population (5, 6).

Dementia is a neurodegenerative disease that involves a decrease in cognitive function and in the ability to perform daily activities. Alzheimer's disease (AD) accounts for 50–70% of dementia and develops progressively, with mood instability and problems with motor function (7). Existing research recognizes the increased risk of falling and fracture in AD patients, attributed to impairment of motor function and balance (8). Moreover, patients with impaired cognition have shown a difficulty in recovering their previous physical independence after fractures when compared to people without cognitive impairment (9). Mild cognitive impairment (MCI) is a transitional stage from aging to AD (10), which worsens to dementia at a rate of 10–15% per year (11). This prodromal stage of AD also involves increased risk of falling (12, 13). Subjects with subjective cognitive decline (SCD) show normal performance in objective cognitive testing but experience cognitive decline (14). SCD is a major area of interest for early prevention of AD in that subjects with SCD have increased risk of further cognitive decline and incidence of AD (15, 16). Risk of falls and fractures increases in SCD (17, 18), although the prevalence is relatively lower than that of MCI and dementia (19). Moreover, cognitive dysfunction, including memory loss, is an intrinsic risk factor for falling and bone mineral density (BMD) decline, contributing to increased fractures in older adults (20, 21).

Despite the significantly increased risk of fractures in normal aging and in the trajectory of AD, there is no alternative to medication and regular screening for fracture. However, there has been growing recognition of the vital effect of physical activity (PA) on delaying and preventing fractures (22). Previous studies have reported that moderate to vigorous intensity PA improves BMD (23), balance, and gait (24) and reduces risk of fall and fracture in older women (25). Moderate to vigorous intensity PA has been reported to decrease risk of fracture in middle aged men (26) and to increase BMD of pelvic and leg bones in older men (27). Additionally, the category of PA evaluated in most prior studies was leisure-time PA, consisting of aerobic, resistance, and balance PAs. Regarding walking, a low-intensity PA, previous research findings have been inconsistent and contradictory. While it has been reported that regular walking reduces the risk of fall in older adults (28), another study has shown that frequent walking is associated with increased fracture risk in people aged 50 years or older (29). Finally, a modifiable effect of PA on fractures has been reported to differ by type of PA (30) and by fracture site (30, 31) in older women.

These previous studies did not categorize the intensity and frequency of PA simultaneously and did not compare various intensities and frequencies of PA for preventing or delaying falls and fractures. Moreover, although the recommended intensity and frequency of PA for decreasing the risk of fall and fracture differ according to target group (32), there are few studies that have evaluated the impact of PA on fracture occurrence in older adults with cognitive decline. This study set out to explore the optimal intensity and frequency of PA to prevent fractures at various sites in cognitively preserved, SCD, and dementia groups of older adults via a large-scale nationwide cohort study. The current paper utilizes a “National Screening Program for Transitional Ages (NSPTA)” database to collectively obtain information on lifestyle, medical history, and cognitive status in the older adults aged 66 years and a database registered with the National Health Insurance service to collect data on fractures and diagnosis of dementia.

## Materials and Methods

### Data Source

The Korean National Health Insurance Service (KNHIS) is a mandatory public health insurance system that provides universal coverage to all residents in South Korea (33). All Koreans who are 40 or older are required, by KNHIS, to receive a compulsory health screening test every 2 years. The National Health Insurance Service-Health Screening Cohort (NHIS-HEALS) participates in this health screening program (34). The NHIS-HEALS includes a health screening called the NSPTA, which was initiated in 2007 for those aged 40 and 66 because they are regarded as middle age and older adults, respectively (35). The NSPTA includes comprehensive questionnaires on medical history, cognitive status, and lifestyle information such as drinking, smoking, and exercising.

Additionally, the NSPTA, which is conducted with the 66-year-old population, contains a questionnaire on subjective cognitive decline (SCD) as assessed by the Prescreening Korean Dementia Screening Questionnaire (KDSQ-P) (36). The KDSQ-P is a 5-item self-reported questionnaire using a 3-Likert type scale (0 for no, 1 for yes, sometimes and 2 for yes, often). The five questions are as follows: Item 1, “Do you think that your memory is worse than that of your peers/friends?”; Item 2, “Do you think your memory is worse than last year?”; Item 3, “Does your memory decline impact important activities/work?”; Item 4, “Do others notice your memory decline?”; and Item 5, “Do you think that you can no longer function as well as before due to your memory decline?.” The total score of the KDSQ-P shows a distribution between 0 and 10, and participants with an overall score of 4 or higher are considered to have significant SCD. The data sources mentioned above are described in detail in previous studies (34, 36, 37).

### Study Cohort

#### Definition of Cognitive Status

**Figure 1:**
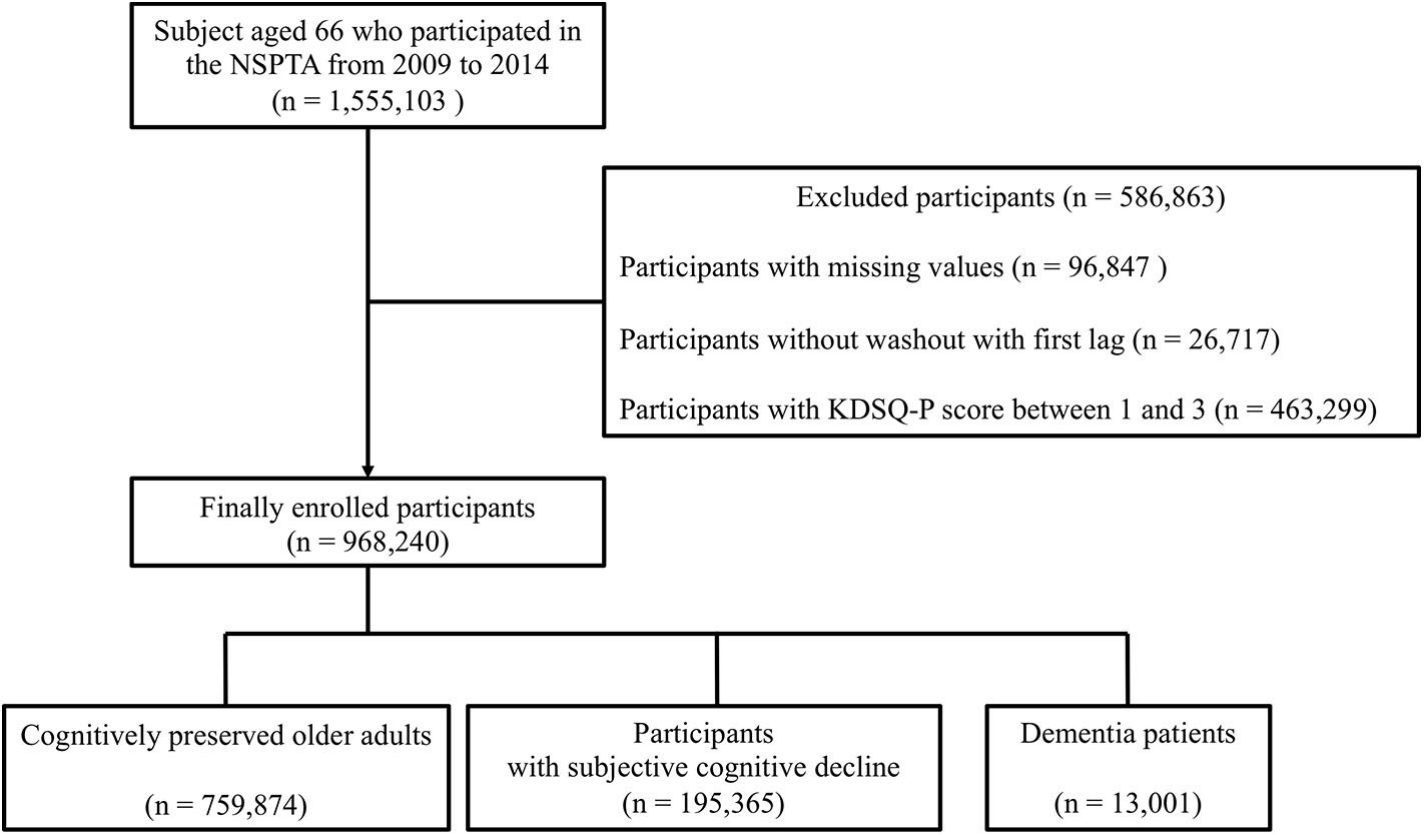
Schematic flow for study population enrollment. NSPTA, National Screening Program for Transitional Ages; KDSQ-P, Prescreening Korean Dementia Screening Questionnaire.

All subjects aged 66 who participated in the NSPTA from 2009 to 2014 were included in the study. Of 1,555,103 NSPTA participants, we excluded those with missing values (*n* = 96,847), those without washout with first lag (*n* = 26,717), and those with KDSQ-P score between 1 and 3 (*n* = 463,299) (Figure 1). A total of 968,240 participants was included in the final analyses. Cognitively preserved participants (CP) (*n* = 759,874) were defined as subjects with a KDSQ-P score of 0, no history of an ICD-10 (the 10th revision of the International Statistical Classification of Diseases and Related Health Problems) dementia code (F00, F01, F02, F03, G30, F051, or G311), and no history of prescription of acetylcholinesterase inhibitors (donepezil, rivastigmine, and galantamine) or NMDA (N-Methyl-D-aspartate) receptor antagonist at the time of NSPTA participation. The SCD subjects (*n* = 195,365) were defined as subjects with KDSQ-P score of 4 or more, because that cut-off point has been reported to be appropriate for detecting a person who needs a screening test for dementia in the validation study of KDSQ-P (36); no history of an ICD-10 dementia code (F00, F01, F02, F03, G30, F051, or G311); and no history of prescription of acetylcholinesterase inhibitors (donepezil, rivastigmine, and galantamine) or NMDA receptor antagonist at the time of NSPTA participation. Dementia (*n* = 13,001) was defined as history or current of prescription of acetylcholinesterase inhibitors or NMDA receptor antagonist with an ICD-10 dementia code (F00, F01, F02, F03, G30, F051, or G311) at the time of NSPTA participation. In the KNHIS, the following criteria must be met for a patient with dementia to receive reimbursement for the prescription of either acetylcholinesterase inhibitors or NMDA receptor antagonist: (1) Mini-mental state examination (MMSE) score of 26 or less and (2) Clinical Dementia Rating (CDR) ≥ 1 or Global Deterioration Scale ≥ 3. Supplementary Materials provide a definition of demographic characteristics and medical history including smoking, alcohol consumption, diabetes, hypertension, dyslipidemia, and fracture history. The definition of medical history has been described in detail in a previous paper (38). The distribution of the sum of KDSQ-P scores in each group is summarized in Supplementary Table 1. This study was approved by the Institutional Review Board of Yeouido St. Mary's Hospital, Seoul, Korea. Consent from individual subjects was not needed because the study used publicly available, anonymous data.

#### Exposure Variable – Physical Activity

The PA was classified into three intensities to provide detailed evidence of implementation. Subjects indicated intensity of PA and the number of days they performed the specific intensity of PA per week in the NSPTA questionnaire. The intensity of PA was classified into vigorous-intensity, moderate-intensity, and walking according to participant self-perception of effort. The questions to confirm subject reply were as follows: (1) vigorous-intensity PA: In the last week, how many days did you perform more than 20 min a day of intense PA that left you much more out of breath than usual? (e.g., running, aerobics, cycling at high speeds, climbing); (2) moderate-intensity PA: In the last week, how many days did you perform a moderate-intensity PA that left you slightly short of breath? (e.g., walking fast, playing doubles tennis, riding a bicycle at normal speed, mopping; excluding the vigorous-intensity PAs); and (3) walking: In the last week, how many days did you walk for more than 30 min a day, totaling at least 10 min at a time? (e.g., light-intensity PA, including commuting or walking during leisure time). However, because subjects can perform different intensities of PA simultaneously in a week, the following definitions were applied to precisely classify the frequency and intensity of PA: (1) Low-frequency & any-intensity PA: subject who performed any of three PAs mentioned above (vigorous-intensity PA, moderate-intensity PA, and walking) fewer than three times a week; (2) High-frequency walking: subject who walks at least three times a week and performs moderate and vigorous-intensity PA fewer than three times a week; (3) High-frequency & moderate-intensity PA: subject who walks fewer than three times a week, performs moderate-intensity PA at least three times a week, and performs vigorous-intensity PA fewer than three times a week; (4) High-frequency & vigorous-intensity PA: subject who walks fewer than three times a week. performs moderate-intensity PA fewer than three times a week, and performs vigorous-intensity PA at least three times a week; (5) High-frequency & multiple-intensity PA: subject who performs any two or all of the three PAs (vigorous-intensity PA, moderate-intensity PA, and walking) at least three times a week. Based on a previous observational study, aerobic and resistance PA at a frequency of 3 times per week were recommended to preserve bone health in adults (32). In this regard, a 'cut-off' frequency of 3 was used to classify the frequency of each PA in the present study.

#### Outcome Variable – Fractures

History of fracture was defined as a hospital visit that resulted in any ICD-10 fracture code within 5 years before the NSPTA. Hip fractures were defined by ICD-10 codes S72.0 and S72.1 within one hospitalization. Vertebral fractures were defined by ICD-10 codes S22.0, S22.1, S32.0, M48.4, and M48.5 within two outpatient clinic visits. Limb fractures were defined as ICD-10 codes for upper arm fractures (S42.0, S42.2, and S42.3), forearm fractures (S52.5 and S52.6), and lower leg fractures (S82.3, S82.5, and S82.6) within two outpatient clinic visits. We explored the impact of frequency and intensity of PA on these categories of fractures based on the adverse consequences and high incidence in older adults (39).

### Statistical Analysis

All continuous variables are expressed as mean ± SD, and categorical data are presented as number (percentage). Study participant characteristics according to cognitive status were compared via one-way analysis of variance (ANOVA) for continuous variables and the x^2^ test for categorical variables. Person-years of follow-up were calculated from the time of NSPTA participation to the occurrence of fracture or to December 31, 2014, whichever came first. Multivariate Cox proportional hazards regression analysis was performed to identify hazard ratios (HRs) of hip, vertebral, and other fractures according to frequency and intensity of PA categorized by low-frequency & any-intensity PA, high-frequency walking, high-frequency & moderate-intensity PA, high-frequency & vigorous-intensity PA, and high-frequency & multiple-intensity PA, with non-PA and low-frequency & any-intensity PA as a reference category. Model 1 was not adjusted; Model 2 was adjusted for age and sex; Model 3 additional was adjusted for income, diabetes, hypertension, dyslipidemia, alcohol consumption, smoking, and BMI; and Model 4 was those plus fracture history. These variables are known risk factors for fracture (40, 41). The proportional hazards assumption was tested for all main effects in all groups. There was no evidence that the proportional hazards assumption was violated in the CP and SCD groups. In the dementia group, the Cox model was a summary of the average situation across the study period. For all statistical analyses, we used SAS version 9.3 (SAS Institute, Cary, NC, USA), with p-values < 0.05 considered significant.

## Results

### Participant Characteristics

A total of 968,540 participants, all aged 66 years, was included in this study. Among them, 759,874 (78.3%), 195,365 (20.2%), and 13,001 (1.5%) were categorized into the CP, SCD, and dementia groups, respectively. Table 1 shows a summary of the baseline characteristics of the study participants. As noted in the Methods, all three groups were the same age (66 years old) and had registered for the NSPTA. The frequencies of vigorous-intensity PA, moderate-intensity PA, and walking in the three groups showed a significant difference. The proportion of subjects who performed PA at a frequency of 1–3 days a week was highest in each group, except for those performed no PA.

**Table 1:** Baseline characteristics of the study population.

	**CP**	**SCD**	**Dementia**	***p***
	**(*n* = 759,874)**	**(*n* = 195,365)**	**(*n* = 13,001)**	
Age (mean ± SD)	66 ± 0	66 ± 0	66 ± 0	
Sex [*n* (% of male)]	375,642 (49.43)	77,062 (39.45)	4,977 (38.28)	<0.0001
Sum of KDSQ-P score (mean ± SD)	0 ± 0	5.07 ± 1.79	3.03 ± 10.34	
Days per week (Vigorous-Int. PA) [*n* (%)]			<0.0001	
0	503160 (66.22)	126625 (64.81)	9791 (75.31)	
1–3	157478 (20.72)	45944 (23.52)	1977 (15.21)	
4–5	49645 (6.53)	12668 (6.48)	570 (4.38)	
6–7	49591 (6.53)	10128 (5.18)	663 (5.1)	
Days per week (Moderate-Int. PA) [*n* (%)]			<0.0001	
0	460,571 (60.61)	113,495 (58.09)	8,876 (68.27)	
1–3	169,140 (22.26)	52,633 (26.94)	2,420 (18.61)	
4–5	64,063 (8.43)	16,224 (8.3)	800 (6.15)	
6–7	66,100 (8.7)	13,013 (6.66)	905 (6.96)	
Days per week (Walking) [*n* (%)]			<0.0001	
0	259404 (34.14)	61057(31.25)	4977(38.28)	
1–3	187061 (24.62)	61613 (31.54)	3318 (25.52)	
4–5	118321 (15.57)	31666 (16.21)	1745 (13.42)	
6–7	195088 (25.67)	41029 (21)	2961 (22.78)	
Smoking [*n* (%)]				<0.0001
None	530,503 (69.81)	138,322 (70.8)	9,895 (76.11)	
Ex-smoker	126,503 (16.65)	34,009 (17.41)	1,863 (14.33)	
Current smoker	102,868 (13.54)	23,034 (11.79)	1,243 (9.56)	
Alcohol Consumption [*n* (%)]			<0.0001	
None	546,041 (71.86)	142,344 (72.86)	11,189 (86.06)	
Mild	129,458 (17.04)	32,546 (16.66)	1,208 (9.29)	
Moderate	48,304 (6.36)	11,050 (5.66)	316 (2.43)	
Heavy	36,071 (4.75)	9,425 (4.82)	288 (2.22)	
Low income [*n* (%)]	227,950 (30)	53,676 (27.47)	4,244 (32.64)	<0.0001
Diabetes [*n* (%)]	156,508 (20.6)	39,794 (20.37)	3,472 (26.71)	<0.0001
Hypertension [*n* (%)]	413,521 (54.42)	101,450 (51.93)	7,409 (56.99)	<00001
Dyslipidemia [*n* (%)]	281,580 (37.06)	76,078 (38.94)	5,949 (45.76)	<0.0001
BMI(kg/m^2^) (mean ± SD)	24.34 ± 3.04	24.24 ± 3.06	24.04 ± 3.28	<0.0001
Fracture history [*n* (%)]	51,567 (6.79)	16,103 (8.24)	1,639 (12.61)	<0.0001

*CP, cognitively preserved older adults; SCD, subjective cognitive decline; Int, intensity; PA, physical activity; BMI, body mass index; SD, standard deviation; p value by ANOVA for continuous variables and by x^2^ test for categorical variables*.

The number of ex-smokers was higher in the SCD group, while current smokers were more numerous in the CP group. The proportion of heavy drinkers was highest in the SCD group. Subjects with low-income level were most frequent in the dementia group. Regarding metabolic risk factors, the proportions of diabetes, hypertension, and hyperlipidemia were concordantly highest in the dementia group. BMI was lowest in the dementia group, although it was generally similar among the three groups. Finally, fracture history was most frequent in the dementia group.

### Effect of Physical Activity on Risk of Fracture

#### Hip Fracture

The incidence rates (number of events per 1,000 person-years) and HRs for hip fractures according to frequency and intensity of PA are demonstrated in Table 2A. In the CP group, all frequencies and intensities of PA reduced the risk of hip fractures compared to non-PA, and high-frequency & vigorous-intensity PA produced the greatest decrease in risk [aHR = 0.497, 95% CI = 0.357–0.691 (Model 4)] compared to non-PA. Compared to low-frequency & any-intensity PA, high-frequency & vigorous-intensity PA only reduced the risk in the CP group [aHR = 0.66, 95% CI = 0.469–0.93 (Model 4)]. In the SCD group, low-frequency & any-intensity PA, high-frequency walking, and high-frequency & multiple-intensity PA reduced the risk of hip fractures compared with that of non-PA. Compared to low-frequency & any-intensity PA, decreased risk was observed only with high-frequency & multiple-intensity PA. In the dementia group, high-frequency walking and high-frequency exercise lowered the risk of hip fracture. In addition, the most noticeable reduction of risk was found for high-frequency & multiple-intensity PA in both the SCD and dementia groups compared with non-PA [SCD, aHR = 0.527, 95% CI = 0.428–0.648 (Model 4); dementia, aHR = 0.4, 95% CI = 0.219–0.733 (Model 4)]. Such reduction was shown for high-frequency & multiple-intensity PA only in the SCD group compared with low-frequency & any-intensity PA [aHR = 0.693, 95% CI = 0.547–0.877 (Model 4)]. Finally, the decrease in risk from non-PA to low-frequency & any-intensity PA was larger than other subsequent changes in frequency and intensity of PA in the CP and SCD groups. Hazard ratios of hip fractures adjusted for Models 1, 2, and 3 are presented in Supplementary Table 2A.

**Table 2:** Effect of physical activity on risk of fracture.

**Group**	**Frequency and intensity of physical activity**	**Number**	**Hip fracture**	**Duration (person years)**	**IR per 1,000**	**Adjusted HR (95% CI) (Model 4): Non-PA (Ref.)**	**Adjusted HR (95% CI) (Model 4): Low-Fre. & Any-Int. PA (Ref.)**
**(A) HIP FRACTURE**							
CP	Non-PA	212,561	841	751,922.08	1.11847	1 (Ref.)	1.329 (1.163, 1.518)
	Low-Fre. & Any-Int. PA	103,842	294	361,750.64	0.81271	0.752 (0.659, 0.86)	1 (Ref.)
	High-Fre. Walking	214,072	657	727,225.55	0.90343	0.812 (0.733,0.9)	1.079 (0.94, 1.238)
	High-Fre. & Moderate-Int. PA	18,923	41	65,907.72	0.62208	0.575 (0.42, 0.787)	0.764 (0.551, 1.059)
	High-Fre. & Vigorous-Int PA	19,116	37	68,472.21	0.54037	0.497 (0.357, 0.691)	0.66 (0.469, 0.93)
	High Fre. & Multiple-Int. PA	191,360	478	648,970.45	0.73655	0.681 (0.608, 0.763)	0.905 (0.782, 1.047)
SCD	Non-PA	49,987	285	193,811.52	1.4705	1 (Ref.)	1.316 (1.076, 1.609)
	Low-Fre. & Any-Int. PA	34,877	144	131,545.03	1.09468	0.76 (0.621, 0.93)	1 (Ref.)
	High-Fre. Walking	53,625	233	197,666.53	1.17875	0.799 (0.671, 0.951)	1.051 (0.854, 1.294)
	High-Fre. & Moderate-Int. PA	4,730	20	17,634.70	1.13413	0.787 (0.5, 1.24)	1.036 (0.649, 1.654)
	High-Fre. & Vigorous-Int PA	4,609	19	17,735.31	1.07131	0.738 (0.463, 1.175)	0.971 (0.602, 1.567)
	High Fre. & Multiple-Int. PA	47,537	134	173,465.70	0.77249	0.527 (0.428, 0.648)	0.693 (0.547, 0.877)
Dementia	Non-PA	4,388	61	12,793.20	4.76816	1 (Ref.)	1.724 (0.977, 3.041)
	Low-Fre. & Any-Int. PA	1,909	15	5,738.28	2.61402	0.58 (0.329, 1.024)	1 (Ref.)
	High-Fre. Walking	3,720	30	11,128.92	2.69568	0.597 (0.384, 0.926)	1.028 (0.553, 1.912)
	High-Fre. & Moderate-Int. PA	272	2	844.85	2.36729	0.527 (0.129, 2.159)	0.908 (0.207, 3.974)
	High-Fre. & Vigorous-Int PA	255	1	790.79	1.26456	0.264 (0.037, 1.907)	0.455 (0.06, 3.447)
	High Fre. & Multiple-Int. PA	2,457	13	7,504.10	1.73239	0.4 (0.219, 0.733)	0.69 (0.328, 1.453)
***p*** **for interaction**						0.1797	0.1797
**(B) VERTEBRAL FRACTURE**							
CP	Non-PA	212,561	6,986	751,922.08	9.2909	1 (Ref.)	1.172 (1.121, 1.225)
	Low-Fre. & Any-Int. PA	103,842	2,734	361,750.64	7.5577	0.853 (0.816, 0.892)	1 (Ref.)
	High-Fre. Walking	214,072	5,471	727,225.55	7.5231	0.833 (0.804, 0.863)	0.977 (0.933, 1.023)
	High-Fre. & Moderate-Int. PA	18,923	441	65,907.72	6.6912	0.77 (0.699, 0.848)	0.902 (0.816, 0.998)
	High-Fre. & Vigorous-Int PA	19,116	392	68,472.21	5.725	0.697 (0.63, 0.772)	0.817 (0.735, 0.909)
	High Fre. & Multiple-Int. PA	191,360	3,853	648,970.45	5.9371	0.713 (0.686, 0.742)	0.836 (0.796, 0.878)
SCD	Non-PA	49,987	2,327	193,811.52	12.0065	1 (Ref.)	1.18 (1.102, 1.263)
	Low-Fre. & Any-Int. PA	34,877	1,286	131,545.03	9.7761	0.847 (0.791, 0.907)	1 (Ref.)
	High-Fre. Walking	53,625	1,914	197,666.53	9.683	0.831 (0.782, 0.883)	0.98 (0.913, 1.052)
	High-Fre. & Moderate-Int. PA	4,730	154	17,634.70	8.7328	0.762 (0.647, 0.897)	0.899 (0.761, 1.063)
	High-Fre. & Vigorous-Int PA	4,609	144	17,735.31	8.1194	0.748 (0.632, 0.885)	0.882 (0.743, 1.048)
	High Fre. & Multiple-Int. PA	47,537	1,285	173,465.70	7.4078	0.681 (0.635, 0.729)	0.803 (0.743, 0.868)
Dementia	Non-PA	4,388	151	12,793.20	11.8031	1 (Ref.)	1.126 (0.837, 1.514)
	Low-Fre. & Any-Int. PA	1,909	62	5,738.28	10.8046	0.888 (0.66, 1.195)	1 (Ref.)
	High-Fre. Walking	3,720	141	11,128.92	12.6697	1.041 (0.827, 1.31)	1.172 (0.869, 1.58)
	High-Fre. & Moderate-Int. PA	272	10	844.85	11.8364	0.896 (0.472, 1.701)	1.009 (0.517, 1.969)
	High-Fre. & Vigorous-Int PA	255	10	790.79	12.6456	1.039 (0.547, 1.972)	1.169 (0.599, 2.282)
	High Fre. & Multiple-Int. PA	2,457	99	7,504.10	13.1928	1.102 (0.854, 1.423)	1.241 (0.903, 1.705)
***p*** **for interaction**						0.0919	0.0919
**(C) LIMB FRACTURE**							
CP	Non-PA	212,561	6,153	751,922.08	8.183	1 (Ref.)	0.997 (0.953,1.042)
	Low-Fre. & Any-Int. PA	103,842	2,836	361,750.64	7.8397	1.003 (0.959, 1.049)	1 (Ref.)
	High-Fre. Walking	214,072	5,689	727,225.55	7.8229	0.969 (0.935, 1.005)	0.966 (0.923, 1.011)
	High-Fre. & Moderate-Int. PA	18,923	495	65,907.72	7.5105	0.974 (0.889, 1.068)	0.971 (0.883, 1.069)
	High-Fre. & Vigorous-Int PA	19,116	452	68,472.21	6.6012	0.923 (0.839, 1.016)	0.92 (0.833, 1.016)
	High Fre. & Multiple-Int. PA	191,360	4,666	648,970.45	7.1898	0.974 (0.938, 1.013)	0.971 (0.927, 1.018)
SCD	Non-PA	49,987	1,865	193,811.52	9.6228	1 (Ref.)	1.049 (0.975, 1.129)
	Low-Fre. & Any-Int. PA	34,877	1,173	131,545.03	8.9171	0.953 (0.886, 1.026)	1 (Ref.)
	High-Fre. Walking	53,625	1,852	197,666.53	9.3693	0.98 (0.919, 1.045)	1.028 (0.956, 1.106)
	High-Fre. & Moderate-Int. PA	4,730	142	17,634.70	8.0523	0.86 (0.725, 1.02)	0.902 (0.758, 1.073)
	High-Fre. & Vigorous-Int PA	4,609	162	17,735.31	9.1343	1.041 (0.886, 1.222)	1.092 (0.927, 1.287)
	High Fre. & Multiple-Int. PA	47,537	1,505	173,465.70	8.6761	0.971 (0.906, 1.04)	1.019 (0.943, 1.099)
Dementia	Non-PA	4,388	125	12,793.20	9.7708	1 (Ref.)	1.106 (0.8, 1.53)
	Low-Fre. & Any-Int. PA	1,909	52	5,738.28	9.0619	0.904 (0.654, 1.25)	1 (Ref.)
	High-Fre. Walking	3,720	110	11,128.92	9.8842	0.981 (0.759, 1.268)	1.085 (0.78, 1.51)
	High-Fre. & Moderate-Int. PA	272	10	844.85	11.8364	1.065 (0.559, 2.03)	1.178 (0.599, 2.319)
	High-Fre. & Vigorous-Int PA	255	6	790.79	7.5874	0.759 (0.334, 1.723)	0.839 (0.36, 1.955)
	High Fre. & Multiple-Int. PA	2,457	87	7,504.10	11.5937	1.166 (0.885, 1.537)	1.29 (0.914, 1.82)
***p*** **for interaction**						0.4722	0.4722

*CP, cognitively preserved older adults; SCD, subjective cognitive decline; Fre., frequency; Int., Intensity; PA, physical activity; Low-frequency & any-intensity PA, subject who performed any of three PAs (vigorous-intensity PA, moderate-intensity PA, and walking) fewer than three times a week; High-Fre. Walking, subject who walks at least three times a week and performs moderate and vigorous-intensity PA fewer than three times a week; High-frequency & moderate-intensity PA, subject who walks fewer than three times a week, performs moderate-intensity PA at least three times a week, and performs vigorous-intensity PA fewer than three times a week; High-frequency & vigorous-intensity PA, subject who walks fewer than three times a week. performs moderate-intensity PA fewer than three times a week, and performs vigorous-intensity PA at least three times a week; High-frequency & multiple-intensity PA, subject who performs any two or all of the three PAs (vigorous-intensity PA, moderate-intensity PA, and walking) at least three times a week; IR, incidence rate; HR, hazard ratio; CI, confidence interval. The results shown are hazard ratios and 95% confidence intervals, with unadjusted HRs (Model 1), HRs adjusted for age and sex (Model 2), additionally adjusted for low income, diabetes mellitus, hypertension, dyslipidemia, alcohol consumption, smoking, and body mass index (Model 3), and adjusted for Model 3 and fracture history (Model 4)*.

#### Vertebral Fracture

The incidence rates and HRs for vertebral fracture according to frequency and intensity of PA are shown in Table 2B. In the CP and SCD groups, all frequencies and intensities of PA reduced the risk of vertebral fractures compared with non-PA. In addition, the most prominent reduction of risk was found in the high-frequency & vigorous-intensity PA in the CP group, compared with both non-PA [aHR = 0.697, 95% CI = 0.63–0.772 (Model 4)] and the low-frequency & any-intensity PA [aHR = 0.817, 95% CI = 0.735–0.909 (Model 4)]. In the SCD group, the high-frequency & multiple-intensity PA produced the greatest decrease in risk compared with both non-PA [aHR = 0.681, 95% CI = 0.635–0.729 (Model 4)] and the low-frequency & any-intensity PA [aHR = 0.803, 95% CI = 0.743–0.868 (Model 4)]. However, the dementia group performing any frequency and intensity of PA did not show decreased risk of vertebral fracture. Lastly, the decrease in risk from non-PA to low-frequency & any-intensity PA was larger than other changes in frequency and intensity of PA in the CP and SCD groups. Hazard ratios of vertebral fractures adjusted for Models 1, 2, and 3 are presented in Supplementary Table 2B.

#### Limb Fracture

The incidence rates and HRs for limb fracture according to frequency and intensity of PA are shown in Table 2C. Any frequency and intensity of PA did not affect the risk of limb fracture in the CP, SCD, and dementia groups (Model 4). Hazard ratios of limb fractures adjusted for Models 1, 2, and 3 are presented in Supplementary Table 2C.

## Discussion

This study evaluated the effects of PA intensity and frequency on the risk of hip, vertebral, and limb fracture in CP, SCD, and dementia groups based on a large-scale nationwide cohort dataset of 66-year-old participants. This study's strength is in comprehensively analyzing the effects of PA on fracture by careful categorization of PA intensity and frequency, cognitive status, and fracture site. Another strength is that this prospective study directly examined the effect of PA on fracture. In contrast, previous studies have mainly evaluated the impact of PA on risk factors such as BMD, fall, and motor function. Lastly, the real-world data of a large sample size provided robust statistical power to evaluate the effects of PA intensity and frequency on fractures at various sites in the course of cognitive impairment development.

The main finding of this study was that the CP participants showed the most prominent decreased risk of hip and vertebral fractures with vigorous-intensity PA at least three times per week compared with low-frequency & any-intensity PA. This finding broadly supports other studies linking vigorous-intensity PA with reduced risk of fall (24–26) and with increased pelvic BMD (27) in older adults. However, the current finding is contrary to a few previous studies showing increased fall risk with vigorous-intensity PA in older men (42) and in older adults with functional limitation (43). Moreover, it has been demonstrated that the effects of vigorous-intensity PA on BMD and bone quality are not significant, despite reducing the risk of fall (44). Therefore, further studies need to be conducted evaluating various indicators for predicting fracture simultaneously.

In vertebral fracture of CP participants, we found reduced risk associated with high-frequency & moderate-intensity PA and high-frequency & multiple-intensity PA. Although there is little research simultaneously comparing the frequency and intensity of PA for preventing fractures, the current findings support clinical evidence that higher levels of PA are associated with decreased risk of recurrent falls in community-dwelling older adults (45). However, the present findings differ from previous research showing that PA decreases the risk of hip fracture but has an inconclusive effect in vertebral fracture (46, 47). Given the lack of assessment of frequency and intensity of PA in previous studies, further work is required to assess the differential effect of PA on fracture according to frequency and intensity.

In the present study, while high-frequency walking alone did not decrease the occurrence of vertebral fracture significantly compared with low-frequency & any-intensity PA, it reduced the risk when performed in conjunction with moderate- or vigorous-intensity PA. Previous studies have reported that walking decreased risk of fracture in older adults (28, 48), but another has shown contrary results that frequent walking increases fracture risk in older adults (29). Therefore, further work considering the frequency of walking and intensity of PA performed in parallel is required to confirm the current findings.

In the SCD group, only high-frequency & multiple-intensity PA reduced the risk of hip and vertebral fractures compared with low-frequency & any-intensity PA. It has been reported that SCD is associated with risk factors such as fall and decreased BMD (19, 20) and primarily increases the risk of hip bone fracture (17, 49). Additionally, it has been suggested that older adults with SCD are vulnerable to fractures (50), and that frailty due to aging could simultaneously increase the risk of fractures and cognitive impairment (51). However, there is little published research on the effects of PA on risk of fracture in older adults with SCD. Furthermore, because few studies have explored differences in prevalence and vulnerability according to fracture site in an SCD group, future studies on these topics are recommended.

Lastly, the decrease in risk of hip and vertebral fractures from non-PA to low-frequency & any-intensity PA was larger than other subsequent changes in frequency and intensity of PA in the CP and SCD groups. Given the current findings, not only high-frequency PA, but also low-frequency PA could have clinical implications in the incidence of fracture in the early trajectory of cognitive decline.

In the dementia group, only high-frequency walking and high-frequency & multiple-intensity PA decreased the risk of hip fracture compared with non-PA. It has been reported that AD patients are at increased risk of falling and hip fracture due to weakened motor function and balance, and that AD and hip fractures share risk factors such as calcium imbalance, vitamin D deficiency, and elevated parathyroid hormone levels (12, 52, 53). Therefore, the vulnerability of motor function and fracture might contribute to the restriction of participation in high-frequency & moderate- and vigorous-intensity PA in the dementia group, which could have affected the small sample size of the higher-intensity PA in the dementia group of the current study. Additionally, it is assumed that the dementia participants who performed high-frequency & multiple-intensity PA had relatively greater preservation of motor function and lower vulnerability to fracture. It is possible that these results merely reflect a selection effect. In addition, participants with dementia had the possibility of a recall bias about intensity and frequency of PA. Therefore, further research should be undertaken to validate the present results.

The present study did not find a significant association between intensity & frequency of PAs and limb fracture risk in any group. Although there are fewer studies on limb fracture than on hip and vertebral fracture, findings have shown no difference in level of physical performance as a physical risk factor for fracture between postmenopausal women with and without radius fracture (54). However, more research needs to be undertaken to understand the association between PA and limb fracture in subjects according to cognitive status.

Several limitations in this study need to be acknowledged. The current study did not evaluate total duration of PA, despite findings that a change in amount of PA affects the risk of fracture (26, 45). In addition, the data in the present study are limited to self-reported PA in the previous week. Furthermore, the current paper did not identify the reasons for fracture or prescription of medication that could affect the risk of fracture. These factors require further caution regarding the generalizability of our findings. Another issue with the current study was the lack of exact classification of PA type. Although previous research has demonstrated that endurance, resistance, and balance exercises reduce the risk of fractures (55), further study is needed to confirm the impact of each PA type on fractures in older adults.

Additionally, given the biomechanical effects of obesity on balance (56) and the linear association between BMI and injury (57), the impact of vigorous-intensity PA on reduced risk of fractures must be analyzed with some caution.

In addition, the frequency and intensity of PA could not be classified in more detail due to a large number of excluded participants; further studies considering these factors are needed. Finally, the scale score for evaluating subjective cognitive change had a wide distribution in the dementia group, possibly because subjects with advanced cognitive impairment are more likely to deny SCD compared with those with less cognitive impairment (58).

This study set out to explore the optimal frequency and intensity of PA to reduce the risk of hip, vertebral, and limb fractures according to cognitive status via a large-scale nationwide cohort dataset. The findings suggest a role of PA according to intensity and frequency in prevention of hip and vertebral fractures in the course of cognitive decline. Further modeling will have to be conducted to verify the effects of PA intensity and frequency on fractures according to cognitive status.

## Data Availability Statement

The datasets generated and analyzed during the current study are available from the corresponding author on reasonable request.

## Ethics Statement

The studies involving human participants were reviewed and approved by An institutional review board of Yeouido St. Mary's Hospital, Seoul, Korea. Written informed consent for participation was not required for this study in accordance with the national legislation and the institutional requirements.

## Author Contributions

DK, S-MW, YU, CL, and HL conceived and designed the research. KH, H-RN, and N-YK collected the data. DK, S-MW, YU, and HL analyzed and interpreted the data. DK and N-YK wrote the initial draft of the manuscript. KH had full access to all the data in the study and take responsibility for the integrity of the data and the accuracy of data analysis. CL and HL provided scientific mentorship throughout the project. All authors contributed to the article and approved the submitted version.

## Conflict of Interest

The authors declare that the research was conducted in the absence of any commercial or financial relationships that could be construed as a potential conflict of interest.
