# Supplementary material for: Differential Risk of Incident Fractures Depending on Intensity and Frequency of Physical Activity According to Cognitive Status: A Nationwide Longitudinal Study
Source: Front Med (Lausanne). 2020 Dec 8;7:572466. doi: 10.3389/fmed.2020.572466 (PMC7753209; doi:10.3389/fmed.2020.572466)
Supplement: Supplementary file 1 [file Data_Sheet_1.docx]

Supplementary Material

**Differential risk of incident fractures depending on intensity and frequency of physical activity according to cognitive status: A Nationwide Longitudinal Study**

Dong Woo Kang^1^, Sheng-Min Wang^2^, Yoo Hyun Um^3^, Hae-Ran Na^2^, Nak-Young Kim^2^, Kyung-do Han^4^, Chang Uk Lee^1^, and Hyun Kook Lim^2^*

^1^Department of Psychiatry, Seoul St. Mary’s Hospital, College of Medicine, The Catholic University of Korea, Seoul, Republic of Korea

^2^Department of Psychiatry, Yeouido St. Mary’s Hospital, College of Medicine, The Catholic University of Korea, Seoul, Republic of Korea

^3^Department of Psychiatry, St. Vincent’s Hospital, College of Medicine, The Catholic University of Korea, Suwon, Republic of Korea

^4^Department of Statistics and Actuarial Science, Soongsil University, Seoul, Republic of Korea

*** Correspondence:**Hyun Kook Lim, MD, PhD

Department of Psychiatry, Yeouido St. Mary’s Hospital, College of Medicine, The Catholic University of Korea, 10, 63-ro, Yeongdeungpo-gu, Seoul, 07345, Republic of Korea

Tel: +82-2-3779-1048, Fax: +82-2-780-6577, E-mail: [drblues@catholic.ac.kr](mailto:drblues@catholic.ac.kr)

**Supplementary Methods and materials**

**1. Definition of demographic characteristics and medical history**

Smoking status was categorized as current smoker, ex-smoker, or never smoked. Data on frequency and amount of alcohol consumed per day were also collected and classified as non-drinker (none), mild drinker (<15g/day), moderate drinker (15.1–29.9 g/day) or heavy drinker (≥ 30 g/day) according to the amount of daily alcohol intake. Low income was defined as participants with an annual income in the lower 20th percentile. The presence of diabetes mellitus was defined according to the presence of ≥1 claim per year under ICD-10 codes E10-14 and ≥1 claim per year for the prescription of anti-diabetic medication or fasting glucose level ≥126 mg/dL. The presence of hypertension was defined according to the presence of ≥1 claim per year under ICD-10 codes I10 or I11 and ≥1 claim per year for the prescription of antihypertensive agents or systolic/diastolic blood pressure ≥140/90 mm Hg. The presence of dyslipidemia was defined according to the presence of ≥1 claim per year under ICD-10 code E78 and ≥1claim per year for the prescription of a lipid-lowering agent or total cholesterol ≥240 mg/dL. Body mass index was calculated as weight in kilograms divided by the square of height in meters.

**Supplementary Table S1. Sum of KDSQ-P score of study population**

|  | **CP (n=759,874)** | **SCD (n=195,365)** | **Dementia (n=13,001)** | ***p*** |
| --- | --- | --- | --- | --- |
| **Sum of KDSQ-P score [n(%)]** |  |  |  | <.0001 |
| 0 | 759,874(100) | 0(0) | 4,566(35.12) |  |
| 1 | 0(0) | 0(0) | 1,213(9.33) |  |
| 2 | 0(0) | 0(0) | 1,206(9.28) |  |
| 3 | 0(0) | 0(0) | 942(7.25) |  |
| 4 | 0(0) | 68,161(34.89) | 887(6.82) |  |
| 5 | 0(0) | 91,092(46.63) | 1,710(13.15) |  |
| 6 | 0(0) | 13,920(7.13) | 457(3.52) |  |
| 7 | 0(0) | 8,102(4.15) | 340(2.62) |  |
| 8 | 0(0) | 5,354(2.74) | 356(2.74) |  |
| 9 | 0(0) | 3,094(1.58) | 294(2.26) |  |
| 10 | 0(0) | 5,642(2.89) | 1,030(7.92) |  |

CP, cognitively preserved older adults; SCD, subjective cognitive decline; KDSQ-P, Prescreening Korean Dementia Screening Questionnaire; *p* value by x^2^ test.

**Supplementary Table S2. Effect of physical activity on risk of fractures (Model 1, 2, and 3)**

**(A) Hip fracture**

| **Group** | **Frequency and Intensity of Physical activity** | **Number** | **Hip Fracture** | **Duration (Person years)** | **IR per 1000** | **Unadjusted HR (95% CI)  (Model 1)** | **Adjusted HR (95% CI) (Model 2)** | **Adjusted HR (95% CI) (Model 3)** |
| --- | --- | --- | --- | --- | --- | --- | --- | --- |
| **CP** | Non-PA | 212,561 | 841 | 751,922.08 | 1.11847 | 1(Ref.) | 1(Ref.) | 1(Ref.) |
|  | Low-Fre. & Any-Int. PA | 103,842 | 294 | 361,750.64 | 0.81271 | 0.728(0.638,0.832) | 0.728(0.637,0.831) | 0.758(0.663,0.866) |
|  | High-Fre. Walking | 214,072 | 657 | 727,225.55 | 0.90343 | 0.817(0.737,0.904) | 0.816(0.737,0.904) | 0.819(0.739,0.907) |
|  | High-Fre. & Moderate-Int. PA | 18,923 | 41 | 65,907.72 | 0.62208 | 0.559(0.409,0.765) | 0.559(0.408,0.764) | 0.582(0.425,0.796) |
|  | High-Fre. & Vigorous-Int PA | 19,116 | 37 | 68,472.21 | 0.54037 | 0.481(0.346,0.669) | 0.481(0.346,0.668) | 0.502(0.361,0.698) |
|  | High Fre. & Multiple-Int. PA | 191,360 | 478 | 648,970.45 | 0.73655 | 0.666(0.595,0.745) | 0.665(0.594,0.744) | 0.689(0.615,0.772) |
| **SCD** | Non-PA | 49,987 | 285 | 193,811.52 | 1.4705 | 1(Ref.) | 1(Ref.) | 1(Ref.) |
|  | Low-Fre. & Any-Int. PA | 34,877 | 144 | 131,545.03 | 1.09468 | 0.749(0.613,0.915) | 0.737(0.603,0.9) | 0.773(0.632,0.946) |
|  | High-Fre. Walking | 53,625 | 233 | 197,666.53 | 1.17875 | 0.811(0.682,0.965) | 0.802(0.675,0.954) | 0.812(0.683,0.967) |
|  | High-Fre. & Moderate-Int. PA | 4,730 | 20 | 17,634.70 | 1.13413 | 0.779(0.495,1.225) | 0.765(0.486,1.204) | 0.803(0.51,1.265) |
|  | High-Fre. & Vigorous-Int PA | 4,609 | 19 | 17,735.31 | 1.07131 | 0.73(0.459,1.162) | 0.699(0.439,1.113) | 0.753(0.472,1.199) |
|  | High Fre. & Multiple-Int. PA | 47,537 | 134 | 173,465.70 | 0.77249 | 0.534(0.435,0.655) | 0.513(0.417,0.631) | 0.537(0.437,0.661) |
| **Dementia** | Non-PA | 4,388 | 61 | 12,793.20 | 4.76816 | 1(Ref.) | 1(Ref.) | 1(Ref.) |
|  | Low-Fre. & Any-Int. PA | 1,909 | 15 | 5,738.28 | 2.61402 | 0.548(0.311,0.964) | 0.549(0.312,0.966) | 0.604(0.342,1.065) |
|  | High-Fre. Walking | 3,720 | 30 | 11,128.92 | 2.69568 | 0.564(0.365,0.874) | 0.564(0.364,0.873) | 0.619(0.399,0.961) |
|  | High-Fre. & Moderate-Int. PA | 272 | 2 | 844.85 | 2.36729 | 0.495(0.121,2.024) | 0.503(0.123,2.056) | 0.582(0.142,2.385) |
|  | High-Fre. & Vigorous-Int PA | 255 | 1 | 790.79 | 1.26456 | 0.263(0.036,1.897) | 0.255(0.035,1.839) | 0.29(0.04,2.095) |
|  | High Fre. & Multiple-Int. PA | 2,457 | 13 | 7,504.10 | 1.73239 | 0.362(0.199,0.659) | 0.355(0.195,0.646) | 0.426(0.233,0.781) |
| ***p* for interaction** |  |  |  |  |  | 0.0361 | 0.1807 | 0.2219 |

**(B) Vertebral fracture**

| **Group** | **Frequency and Intensity of Physical activity** | **Number** | **Vertebral  Fracture** | **Duration (Person years)** | **IR per 1000** | **Unadjusted HR (95% CI)  (Model 1)** | **Adjusted HR (95% CI) (Model 2)** | **Adjusted HR (95% CI) (Model 3)** |
| --- | --- | --- | --- | --- | --- | --- | --- | --- |
| **CP** | Non-PA | 212,561 | 6,986 | 751,922.08 | 9.2909 | 1(Ref.) | 1(Ref.) | 1(Ref.) |
|  | Low-Fre. & Any-Int. PA | 103,842 | 2,734 | 361,750.64 | 7.5577 | 0.814(0.779,0.851) | 0.848(0.812,0.887) | 0.859(0.822,0.898) |
|  | High-Fre. Walking | 214,072 | 5,471 | 727,225.55 | 7.5231 | 0.816(0.787,0.845) | 0.833(0.804,0.863) | 0.84(0.811,0.871) |
|  | High-Fre. & Moderate-Int. PA | 18,923 | 441 | 65,907.72 | 6.6912 | 0.722(0.656,0.795) | 0.764(0.694,0.841) | 0.778(0.706,0.856) |
|  | High-Fre. & Vigorous-Int PA | 19,116 | 392 | 68,472.21 | 5.725 | 0.615(0.555,0.681) | 0.69(0.624,0.764) | 0.705(0.636,0.78) |
|  | High Fre. & Multiple-Int. PA | 191,360 | 3,853 | 648,970.45 | 5.9371 | 0.644(0.619,0.669) | 0.708(0.68,0.736) | 0.722(0.694,0.751) |
| **SCD** | Non-PA | 49,987 | 2,327 | 193,811.52 | 12.0065 | 1(Ref.) | 1(Ref.) | 1(Ref.) |
|  | Low-Fre. & Any-Int. PA | 34,877 | 1,286 | 131,545.03 | 9.7761 | 0.818(0.764,0.876) | 0.846(0.79,0.905) | 0.862(0.805,0.923) |
|  | High-Fre. Walking | 53,625 | 1,914 | 197,666.53 | 9.683 | 0.814(0.766,0.865) | 0.833(0.784,0.885) | 0.844(0.795,0.897) |
|  | High-Fre. & Moderate-Int. PA | 4,730 | 154 | 17,634.70 | 8.7328 | 0.733(0.623,0.863) | 0.759(0.645,0.893) | 0.776(0.659,0.913) |
|  | High-Fre. & Vigorous-Int PA | 4,609 | 144 | 17,735.31 | 8.1194 | 0.678(0.573,0.802) | 0.741(0.626,0.877) | 0.761(0.643,0.901) |
|  | High Fre. & Multiple-Int. PA | 47,537 | 1,285 | 173,465.70 | 7.4078 | 0.624(0.583,0.668) | 0.677(0.632,0.724) | 0.694(0.648,0.744) |
| **Dementia** | Non-PA | 4,388 | 151 | 12,793.20 | 11.8031 | 1(Ref.) | 1(Ref.) | 1(Ref.) |
|  | Low-Fre. & Any-Int. PA | 1,909 | 62 | 5,738.28 | 10.8046 | 0.913(0.679,1.227) | 0.909(0.676,1.222) | 0.908(0.675,1.221) |
|  | High-Fre. Walking | 3,720 | 141 | 11,128.92 | 12.6697 | 1.07(0.851,1.346) | 1.071(0.851,1.347) | 1.07(0.85,1.347) |
|  | High-Fre. & Moderate-Int. PA | 272 | 10 | 844.85 | 11.8364 | 0.997(0.526,1.892) | 0.968(0.511,1.837) | 0.973(0.512,1.846) |
|  | High-Fre. & Vigorous-Int PA | 255 | 10 | 790.79 | 12.6456 | 1.065(0.562,2.02) | 1.134(0.598,2.151) | 1.139(0.6,2.163) |
|  | High Fre. & Multiple-Int. PA | 2,457 | 99 | 7,504.10 | 13.1928 | 1.113(0.864,1.434) | 1.159(0.899,1.493) | 1.161(0.899,1.498) |
| ***p* for interaction** |  |  |  |  |  | 0.7853 | 0.0402 | 0.0405 |

**(C) Limb fracture**

| **Group** | **Frequency and Intensity of Physical activity** | | | **Number** | **Limb  Fracture** | **Duration (Person years)** | **IR per 1000** | **Unadjusted HR (95% CI)  (Model 1)** | **Adjusted HR (95% CI) (Model 2)** | **Adjusted HR (95% CI) (Model 3)** |
| --- | --- | --- | --- | --- | --- | --- | --- | --- | --- | --- |
| **CP** | | Non-PA | | 212,561 | 6,153 | 751,922.08 | 8.183 | 1(Ref.) | 1(Ref.) | 1(Ref.) |
|  |  | Low-Fre. & Any-Int. PA | | 103,842 | 2,836 | 361,750.64 | 7.8397 | 0.958(0.916,1.001) | 1.007(0.963,1.052) | 1.01(0.966,1.056) |
|  |  | High-Fre. Walking | | 214,072 | 5,689 | 727,225.55 | 7.8229 | 0.955(0.922,0.99) | 0.98(0.946,1.016) | 0.977(0.943,1.013) |
|  |  | High-Fre. & Moderate-Int. PA | | 18,923 | 495 | 65,907.72 | 7.5105 | 0.918(0.837,1.006) | 0.982(0.896,1.077) | 0.985(0.899,1.079) |
|  |  | High-Fre. & Vigorous-Int PA | | 19,116 | 452 | 68,472.21 | 6.6012 | 0.807(0.733,0.888) | 0.93(0.845,1.023) | 0.932(0.847,1.026) |
|  |  | High Fre. & Multiple-Int. PA | | 191,360 | 4,666 | 648,970.45 | 7.1898 | 0.878(0.845,0.912) | 0.985(0.948,1.024) | 0.986(0.949,1.025) |
| **SCD** | | Non-PA | | 49,987 | 1,865 | 193,811.52 | 9.6228 | 1(Ref.) | 1(Ref.) | 1(Ref.) |
|  |  | Low-Fre. & Any-Int. PA | | 34,877 | 1,173 | 131,545.03 | 8.9171 | 0.926(0.861,0.996) | 0.964(0.896,1.037) | 0.969(0.901,1.043) |
|  |  | High-Fre. Walking | | 53,625 | 1,852 | 197,666.53 | 9.3693 | 0.973(0.912,1.037) | 0.999(0.937,1.066) | 0.996(0.934,1.062) |
|  |  | High-Fre. & Moderate-Int. PA | | 4,730 | 142 | 17,634.70 | 8.0523 | 0.836(0.705,0.992) | 0.871(0.734,1.033) | 0.875(0.737,1.037) |
|  |  | High-Fre. & Vigorous-Int PA | | 4,609 | 162 | 17,735.31 | 9.1343 | 0.949(0.809,1.115) | 1.054(0.898,1.238) | 1.061(0.903,1.246) |
|  |  | High Fre. & Multiple-Int. PA | | 47,537 | 1,505 | 173,465.70 | 8.6761 | 0.901(0.842,0.964) | 0.991(0.925,1.061) | 0.99(0.925,1.06) |
| **Dementia** | | Non-PA | | 4,388 | 125 | 12,793.20 | 9.7708 | 1(Ref.) | 1(Ref.) | 1(Ref.) |
|  |  | Low-Fre. & Any-Int. PA | | 1,909 | 52 | 5,738.28 | 9.0619 | 0.928(0.672,1.283) | 0.924(0.669,1.277) | 0.927(0.67,1.282) |
|  |  | High-Fre. Walking | | 3,720 | 110 | 11,128.92 | 9.8842 | 1.012(0.783,1.308) | 1.012(0.783,1.308) | 1.008(0.78,1.303) |
|  |  | High-Fre. & Moderate-Int. PA | | 272 | 10 | 844.85 | 11.8364 | 1.21(0.636,2.305) | 1.174(0.617,2.237) | 1.143(0.6,2.179) |
|  |  | High-Fre. & Vigorous-Int PA | | 255 | 6 | 790.79 | 7.5874 | 0.776(0.342,1.76) | 0.828(0.365,1.879) | 0.822(0.362,1.866) |
|  |  | High Fre. & Multiple-Int. PA | | 2,457 | 87 | 7,504.10 | 11.5937 | 1.187(0.903,1.561) | 1.237(0.941,1.627) | 1.222(0.927,1.61) |
| ***p* for interaction** | | |  |  |  |  |  | 0.264 | 0.4169 | 0.4021 |

CP, cognitively preserved older adults; SCD, subjective cognitive decline; Fre., frequency; Int., Intensity; PA, physical activity; Low-frequency & any-intensity PA, subject who performed any of three PAs (vigorous-intensity PA, moderate-intensity PA, and walking) fewer than three times a week; High-Fre. Walking, subject who walks at least three times a week and performs moderate and vigorous-intensity PA fewer than three times a week; High-frequency & moderate-intensity PA, subject who walks fewer than three times a week, performs moderate-intensity PA at least three times a week, and performs vigorous-intensity PA fewer than three times a week; High-frequency & vigorous-intensity PA, subject who walks fewer than three times a week. performs moderate-intensity PA fewer than three times a week, and performs vigorous-intensity PA at least three times a week; High-frequency & multiple-intensity PA, subject who performs any two or all of the three PAs (vigorous-intensity PA, moderate-intensity PA, and walking) at least three times a week; IR, incidence rate; HR, hazard ratio; CI, confidence interval. The results shown are hazard ratios and 95% confidence intervals, with unadjusted HRs (Model 1), HRs adjusted for age and sex (Model 2), additionally adjusted for low income, diabetes mellitus, hypertension, dyslipidemia, alcohol consumption, smoking, and body mass index (Model 3)
